# Magnetosensation during re-learning walks in desert ants (*Cataglyphis nodus*)

**DOI:** 10.1007/s00359-021-01511-4

**Published:** 2021-10-22

**Authors:** Pauline N. Fleischmann, Robin Grob, Wolfgang Rössler

**Affiliations:** Behavioral Physiology and Sociobiology (Zoology II), Biocenter, University of Würzburg, 97074 Würzburg, Germany

**Keywords:** Landmark panorama, Learning and memory, Magnetic compass, Navigation, Path integration

## Abstract

At the beginning of their foraging careers, *Cataglyphis* desert ants calibrate their compass systems and learn the visual panorama surrounding the nest entrance. For that, they perform well-structured initial learning walks. During rotational body movements (pirouettes), naïve ants (novices) gaze back to the nest entrance to memorize their way back to the nest. To align their gaze directions, they rely on the geomagnetic field as a compass cue. In contrast, experienced ants (foragers) use celestial compass cues for path integration during food search. If the panorama at the nest entrance is changed, foragers perform re-learning walks prior to heading out on new foraging excursions. Here, we show that initial learning walks and re-learning walks are structurally different. During re-learning walks, foragers circle around the nest entrance before leaving the nest area to search for food. During pirouettes, they do not gaze back to the nest entrance. In addition, foragers do not use the magnetic field as a compass cue to align their gaze directions during re-learning walk pirouettes. Nevertheless, magnetic alterations during re-learning walks under manipulated panoramic conditions induce changes in nest-directed views indicating that foragers are still magnetosensitive in a cue conflict situation.

## Introduction

The geomagnetic field (GMF) offers useful information for animal orientation across taxa. Many animal species rely on the GMF for migration purposes (for a review: Mouritsen 2018), most famously birds (for a review: Wiltschko and Wiltschko 2005) and sea turtles (e.g. Lohmann et al. 2004), but also fishes (salmon: e.g. Putman et al. 2020; Quinn 1980) and arthropods (lobster: Boles and Lohmann 2003; bogong moth: Dreyer et al. 2018; monarch butterfly: Guerra et al. 2014). However, the GMF may also be a useful cue for close-range orientation (Wyeth 2010). Hymenoptera, such as honey bees and desert ants, use the GMF for non-migratory navigational tasks (for a review: Fleischmann et al. 2020). *Cataglyphis nodus* ants use the GMF as a reference system to align their gaze directions towards the nest entrance during their initial learning walks (iLWs) at the beginning of their foraging careers (Fleischmann et al. 2018a). The ants’ use of the GMF for path integration (PI) under natural conditions during iLWs represents the only example for the role of the magnetic sense in a specific navigation task in insects (for definition of “navigation”, see Grob et al. (2021)) with all other potential cues for orientation available. Since *Cataglyphis* ants are well-known for navigation by means of PI using a celestial compass system during foraging (for a review: Wehner 2020), a puzzling question is when, why and how ants switch from using the GMF as a compass cue to using celestial cues, such as the sun’s position or the UV polarization pattern of the sky.

*Cataglyphis* ants are highly skilled navigators using a wide range of orientation cues (for a review: Wehner 2020). Their navigational capacities are especially impressive, because the ants undergo an age-related division of labor. For most of their lives they work in the dark nest before navigating mainly visually (Schmid-Hempel and Schmid-Hempel 1984). After eclosion, the ants’ cuticle is still soft and pale. After this “callow” stage, ants enter the interior I stage during which they serve as motionless food storages for the colony. Afterwards, ants perform maintenance tasks within the nest, e.g. caring for the brood and the queen, or digging (interior II stage). Only then, after weeks in the darkness of the nest, novices leave the nest entrance to become foragers. *Cataglyphis* ants use up to 3 days to perform learning walks (LWs) to acquire all information for orientation during foraging far away from the nest (Fleischmann et al. 2016, 2018b; Stieb et al. 2012; Wehner et al. 2004; for a review: Zeil and Fleischmann 2019). The transition from interior to exterior worker is not only characterized by changes in behavior, but also leads to neuroplastic changes along visual pathways in the ant brain (Grob et al. 2017; Habenstein et al. 2021; Schmitt et al. 2016; Stieb et al. 2012; for reviews: Grob et al. 2019; Rössler 2019). The iLWs include pirouettes, rotational body movements that are full or partial turns during which the novices stop several times (Fleischmann et al. 2017). During the longest stopping phase, they gaze back to the nest entrance, a tiny hole in the ground invisible from the ant’s perspective. Most importantly, novices do not use celestial cues as reference system for aligning their gaze directions (Grob et al. 2017), but the GMF (Fleischmann et al. 2018a).

Experienced *Cataglyphis* foragers usually leave the nest fast to search for food to then return to the nest following their so-called home vector (Müller and Wehner 1988). Ants perform re-learning walks (reLWs) after substantial portions of the panorama around the nest entrance have been changed (*Cataglyphis fortis*: Fleischmann et al. 2016; Vega Vermehren et al. 2020; *Ocymyrmex robustior*: Müller and Wehner 2010; *Myrmecia croslandi*: Jayatilaka et al. 2018; *Myrmecia pyriformis*: Narendra and Ramirez-Esquivel 2017; for a review: Zeil and Fleischmann 2019) or before they start to return to their nest after visiting a new feeder (*Formica* rufa: Nicholson et al. 1999). *Cataglyphis*’ iLWs and reLWs show clear similarities and differences, but until now the structures of iLWs and of reLWs have never been compared systematically. One obvious similarity is that both LW types include pirouettes (this study). LW pirouettes offer an ideal behavioral read out, because the gaze direction of the ant during the longest stopping phase within a pirouette indicates, where the ant expects its nest entrance (Fleischmann et al. 2017).

The first goal of our study was to compare the structure of iLWs and reLWs in detail. For that we recorded all outbound trips (iLWs, foraging trips and reLWs) of individually marked ants at the nest entrance and compared the structure of iLWs and reLWs of the very same ants at different stages of their foraging career (novices and experienced foragers). Since both types of LWs included pirouettes, we additionally aimed testing whether foragers rely on the GMF for aligning their gaze directions during reLW pirouettes, comparable to the behavior of novices during their iLWs. For that, we turned the horizontal component of the GMF using a Helmholtz coil and recorded reLWs of experienced foragers before and after magnetic alterations. Our results show that iLWs and reLWs show structural differences, and that novices and foragers rely on different reference systems to align their gaze directions during pirouettes in iLWs and reLWs, respectively.

## Materials and methods

### Test animals and study site

Experiments were performed with *Cataglyphis nodus* ants (Brullé 1832) in June and July 2019 in Schinias National Park (Marathon, Greece). We used two nests located at different clearings in the surrounding pine forest for the two experiments. Trees as natural landmarks provided a prominent panorama. All ants outside the nest were marked with one color (Motip Lackstift Acryl, MOTIP DUPLI GmbH, Haßmersheim, Germany) for 3 days before the actual experiments started. Test ants were multi-colored.

### Experiment 1: Comparison of initial learning walks and re-learning walks

We performed this experiment to compare the characteristics of iLWs with reLWs at different life stages of the very same ants. For that, we monitored the life histories of individually marked ants from their first appearance outside the nest until they became experienced foragers. After the 3 day marking period (401 exterior ants) prior to the experiment, all unmarked ants were considered to be naïve (“novices”). We caught 33 novices and marked them individually with a unique two-dot color code. Every time one of these individually marked ants left the nest or returned to the nest, we started a video recording. At noon of the fifth experimental day, when the majority of individually marked ants had been observed to bring back food (“foragers”), we placed an additional landmark (white cylinder: 50 cm height, 9 cm diameter) in the recording area (40 cm southeast of the nest entrance) to trigger reLWs. These non-stop observations resulted in a logbook with 783 entries documenting the iLWs, reLWs and outbound as well as inbound foraging trips of the individually marked foragers. Ten of the 33 initially marked ants re-appeared on the afternoon when the landmark was installed to induce reLWs.

### Experiment 2 A and 2B: Magnetosensation of experienced foragers

**Fig. 1:**
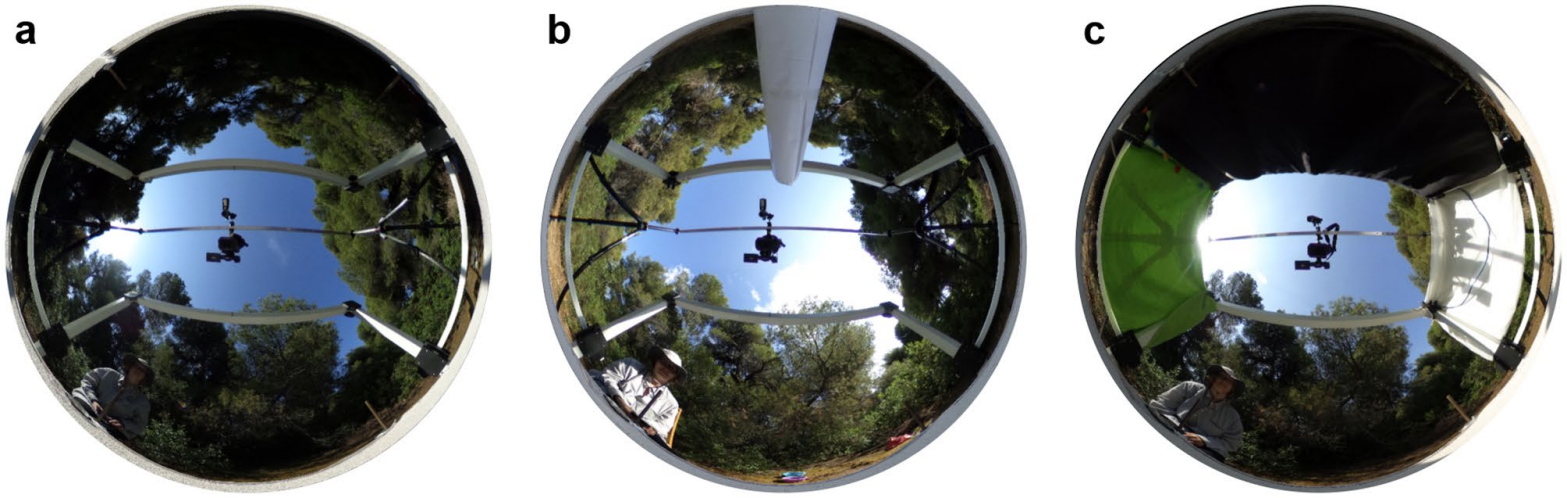
Setup from the ant’s perspective. **a** Helmholtz coil and camera setup as it was installed during training. **b** Setup with additional landmark in the north. **c** Setup with new panorama represented by three large sheets (black—north, green—east, white—west). The observer was always sitting in the south–east.

We performed the second experiment to test whether experienced foragers use the geomagnetic field to align their gaze directions during reLW pirouettes as novices do during their iLWs (Fleischmann et al. 2018a). For that, we confronted approved foragers (116 ants of 241 ants marked outside the nest were observed to bring back food items) with a changed panorama around the nest to trigger reLWs, and altered the geomagnetic field using a Helmholtz coil (Fig. 1a). We performed two variants of this experiment, (A) with a single additional landmark (white cylinder: 50 cm height, 9 cm diameter) in the recording area (30 cm north of the artificial nest entrance on the experimental platform) (Fig. 1b), and (B) with a completely new panorama (three large sheets in green (east), white (west) and black (north)) (Fig. 1c).

Prior to the actual experiment, we additionally marked ants that brought food back to the nest with a second color (“foragers”). Only approved foragers, i.e., ants that had been marked with two different colors, participated in the experiment and were video recorded. When a forager left the nest for a foraging trip, we started the video recording. When the test ant had performed at least one reLW pirouette, we switched on the Helmholtz coil system causing a 180° rotation of the horizontal component of the magnetic field. The Helmholtz coil system was switched off and the video recording was stopped, when the forager had performed at least one pirouette under altered magnetic conditions or had exited the recording area by returning to the nest or by leaving the experimental platform.

To experimentally alter the magnetic field, we used our established Helmholtz coil setup (Fleischmann et al. 2018a) consisting of a rectangular Helmholtz coil (HHS 5213-100, Schwarzbeck Mess-Elektronik, Schönau, Germany), a customized DC power supply, and an elevated platform. Since the homogeneity of the magnetic field is highest in the center of the coil system, ants were trained to leave the nest via the platform (60 cm × 60 cm). The natural nest entrance was covered with nest cover (a cylindrical box with a tunnel), so that the ants could still leave their nest, but had to use this artificial nest entrance (diameter 3 cm). We installed the experimental setup (Helmholtz coil, cameras and platform) every morning, and removed it every evening. The setup was north-oriented and leveled. During the experiment, the tunnel of the nest cover was connected with the platform via a flexible tube. Ants left the tube through the artificial nest entrance (diameter: 3 cm) in the center of the platform. They could leave the platform by walking on one of four fabric ramps. Foragers quickly learned to use these ramps for their outbound and inbound trips. In the morning, we measured the natural geomagnetic field and the experimental magnetic field by the Helmholtz coil with a magnetometer (MEDA FVM400 vector magnetometer, Inc. Macintyre Electronic Design Associates, Inc. 43676 Trade Center Place, Suite 145 Dulles, VA 20166). At the nest (N 38° 08.636′, E 024° 02.041′) used for this experiment, the GMF and the experimentally altered magnetic field had the following characteristics (Experiment 2 A/B). Under natural conditions (geomagnetic field), the inclination was *I* = 54.3°/54.8° and total intensity was *B* = 46.270 µT/46.269 µT (with the components *X* = 26.889 µT/26.628 µT, *Y* = − 0.114 µT/0.198 µT, *Z* = 37.656 µT/37.834 µT). Under experimental conditions (Helmholtz coil on), the inclination was *I* = 54.9°/55.1° and the total intensity *B* = 46.593 µT/46.526 µT (with the components *X* = − 26.779µT/− 26.573, *Y* = 0.662 µT/0.662 µT, *Z* = 38.119 µT/38.182 µT).

### Video recordings and analysis

We installed two cameras above the recording area (i.e., above the natural nest entrance in experiment 1, or above the platform in experiment 2) to record the ants’ paths. A camcorder (HDR-CX330E, Sony Corporation, Minato, Japan) recorded the experimental area nonstop during the experiments with 25 fps and full-HD. A 4K camcorder (HC-X1000, Panasonic Corporation, Kadoma, Japan) recorded the paths of the test ants at 50 fps. Every time a test ant left the nest entrance, the observer sitting next to nest started the recording of the 4K camcorder using the Panasonic Image App (Version 10.9.2, Panasonic Corporation, Kadoma, Japan) on a Cat S60 smartphone (Caterpillar, Peoria, USA). Video recordings were stopped when the ant returned to the nest or left the recording area. In experiment 1, video recordings were also started when an individually marked test ant returned from a foraging trip and entered the recording area from outside.

We converted the 4K videos into image stacks using the Free Video to JPG Converter (v. 5.0.101 build 201, DVDVideoSoft, DIGITAL WAVE LTD., London, UK). We analyzed the ants’ paths manually frame by frame using the MATLAB (2015a, MathWorks, Natick, MA, USA) application DIGILITE (Jan Hemmi and Robert Parker, The Australian National University, Canberra, Australia). For that, we marked the positions of thorax and mandibles in each frame. In addition, the nest entrance position was marked.

To determine the gaze directions relative to the nest during LW pirouettes, we used the coordinates of mandibles, thorax and nest entrance as has been established before (Fleischmann et al. 2017, 2018a; Grob et al. 2017). The gaze direction to the nest was defined as 180° (angle between mandibles-nest and mandibles-thorax). The relative gaze direction during the longest stopping phase (minimal duration: 100 ms) of each pirouette was used for statistical analyses (Fleischmann et al. 2017, 2018a; Grob et al. 2017). In experiments 2 A and 2B, where the magnetic field was rotated by 180°, the fictive nest entrance position was calculated for each ant (Fleischmann et al. 2018a). For that, the mandibles-nest vector was rotated by 180° when the magnetic field has been experimentally rotated pointing towards the fictive nest entrance position (Fig. 2b in Fleischmann et al. 2018a). We analyzed data of the pirouettes after the Helmholtz coil was switched on both relative to the nest entrance and relative to the fictive nest entrance position.

### Statistics

To analyze the circular data, we used Oriana 4.02 (Kovach Computing Services, Anglesey, UK). Gaze directions during the longest stopping phases of pirouettes were grouped into 10°-bins for plotting. We performed the Rayleigh Uniformity Test to check whether the data sets were uniformly distributed or differed significantly from a uniform distribution (significance level: 0.05, 0.01, 0.001). If possible, we calculated the mean vector (*µ*), and the 95% confidence interval (95% CI) to check whether the expected direction (nest entrance or fictive nest entrance defined as 180°) lay within the interval limits. To compare distributions (experiment 1: iLWs versus reLWs, experiment 2: before magnetic alteration versus after magnetic alteration), we used pairwise Mardia–Watson–Wheeler test (significance level: 0.05, 0.01, 0.001). To compare the proportions of ants returning to the nest with those leaving the recording area during iLWs and reLWs, respectively, we performed Fisher’s Exact Test (two-sided, significance level: *0.05, **0.01, ***0.001).

## Results

### Characterization of re-learning walks

**Fig. 2:**
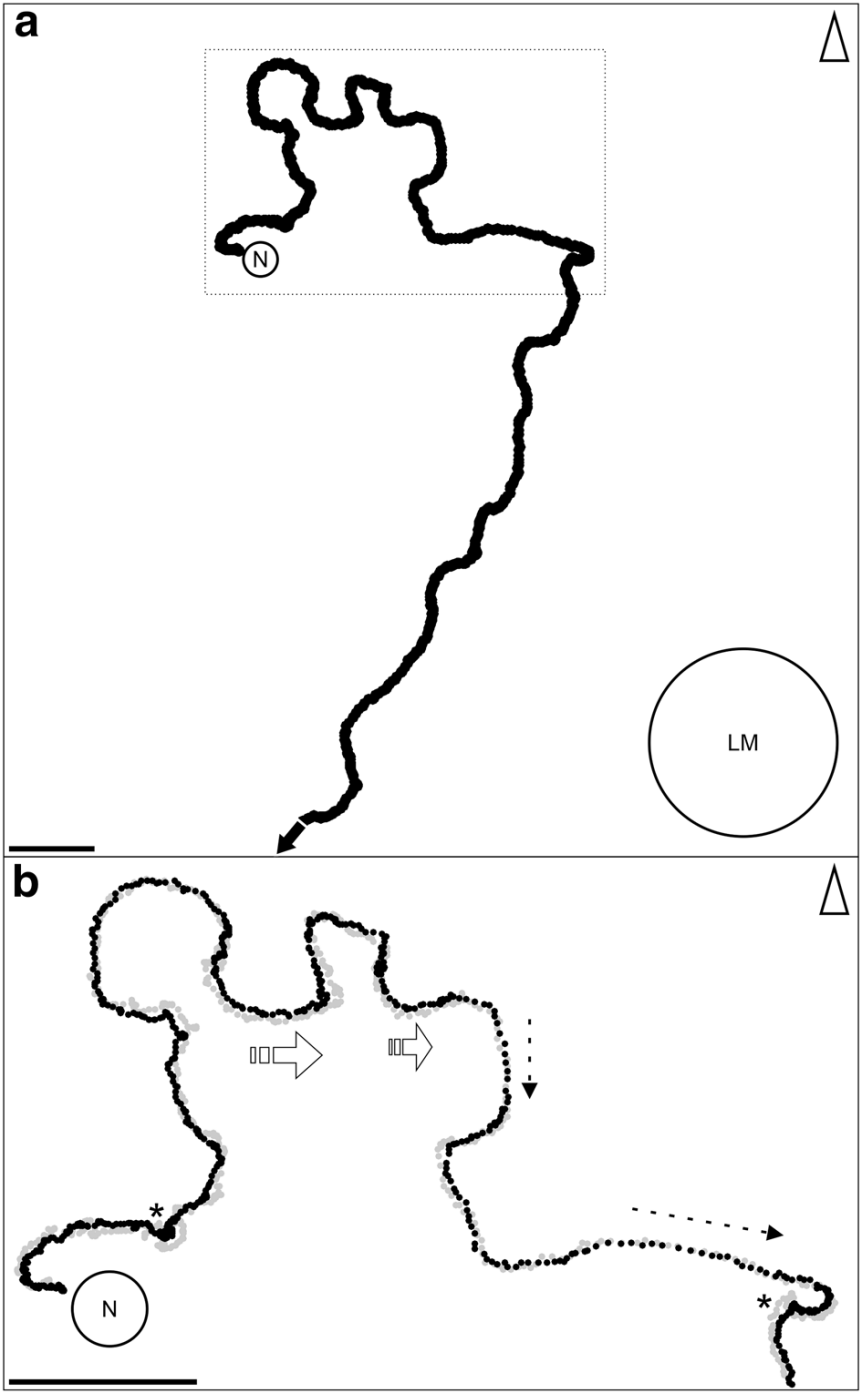
Characteristics of re-learning walks (reLWs). **a** Individual path of a reLW (black line) performed by a forager leaving the nest (N) shows that after the introduction of an artificial landmark (LM) the ant moves towards the opposite direction (north) following a meandering path before leaving the recording area along a more straight path towards south (black arrow). The dotted rectangle marks the area that is shown in more detail in **b**. **b** Both traces of the mandibles (gray) and of the thorax (black) reveal the fine structure of the reLW. The forager performed pirouettes (indicated by stars *), tight turns about the body axis. During meandering it moved laterally (gray and black dots lie parallel to each other, white arrows indicate the direction of movement). The reLWs also includes straight path segments (gray and black dots lie above each other, dotted arrows indicate the direction of movement) during which the forager moved faster (dots in the path represent the positions of the mandibles (gray) and the thorax (black) every 20 ms). The triangles point towards north. Scale bars = 5 cm

We introduced an artificial landmark next to the natural nest entrance to induce reLWs in approved foragers. On the first nest departure after the new landmark was introduced, foragers typically circled around the nest entrance before leaving the nest area to forage (Fig. 2a). Usually, they started their trips into the opposite direction of where they pursued the foraging trips, and they entered almost every sector around the nest entrance before running off. Foragers’ reLWs included different elements (Fig. 2b). We observed pirouettes that are partial or full turns about the ant’s body axis (cf. definition for pirouettes in iLWs of novices (Fleischmann et al. 2017)). Furthermore, experienced ants showed a behavior that we called “meandering”. When foragers meandered, they systematically explored the surrounding. The ant’s path typically showed several curves. In addition, during meandering the ants moved laterally, i.e., gaze direction and walking direction were not the same. Straight path segments during which the ants moved fast connected these elements.

**Fig. 3:**
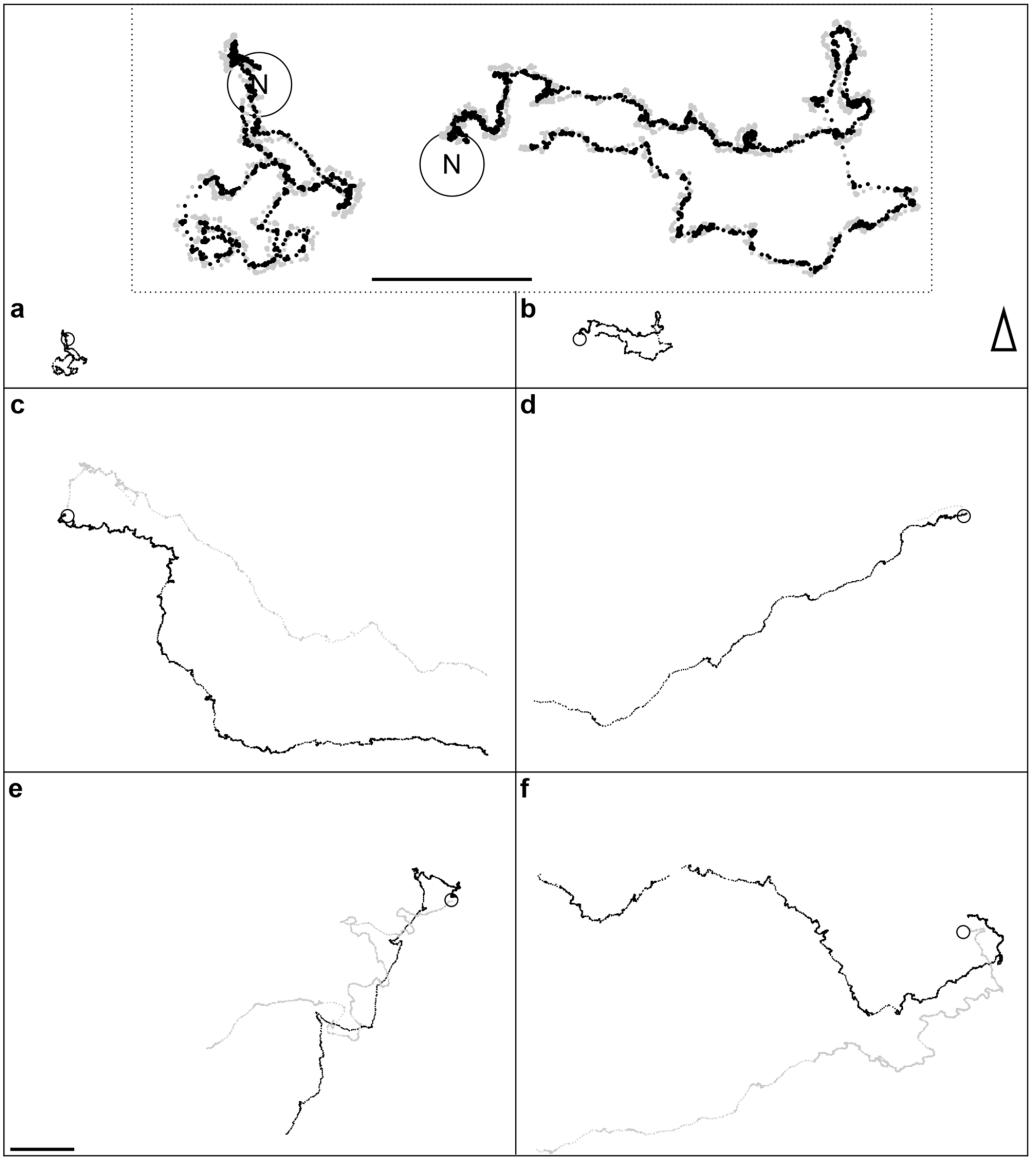
Comparison of the structures of initial learning walks (iLWs) and of re-learning walks (reLWs). Examples of paths from one individually marked ant at different stages along its lifetime. **a**, **b** First (30.6 s) and second (44.2 s) iLWs at the beginning of the ant’s foraging career. The ant was marked 1 day before reappearing and starting to perform short learning walks during which it explored different sectors around the nest entrance (first south sector, then east sector). Before leaving the nest entrance (open circle) for the learning walks, the ant looked out from the nest entrance several times (two times before the first and two times before the second learning walk). The time between the first and second learning walk was 15 min. The inset above **a** and **b** shows the ant’s paths at higher magnification. Positions of the mandibles (gray) and of the thorax (black) are marked separately every 20 ms. **c–f** Outbound (black) and inbound (gray) path of the ant’s foraging trips. **c** First foraging trip performed 2 days after the first learning walks. **d** As an experienced forager, the ant left the nest in a straight line and returned very fast (we detected the returning ant just shortly before entering the nest; therefore, the inbound path was only recorded during the last few centimeters). **e**, **f** First and second reLW after setup of the landmark. The paths document the typical characteristics of reLWs. The ant left the recording area to pursue a foraging trip after circling around the nest entrance. It turned several times and meandered during both outbound and inbound trip. Note that in **f** the outbound path is missing a short part of the path, because the ants run below the camera’s tripod leg. The ant’s position (thorax) was marked every 20 ms. The triangles point towards north. Scale bars = 5 cm

**Fig. 4:**
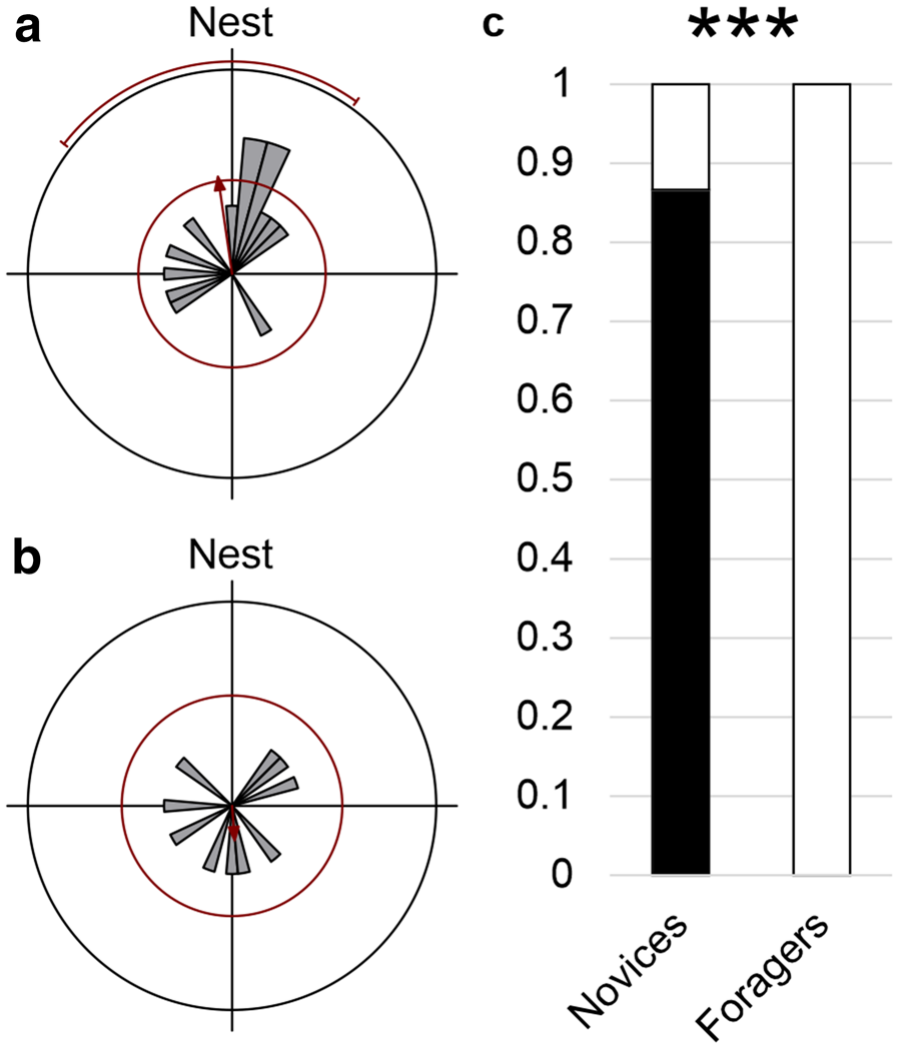
Differences between initial learning walks (iLWs) and re-learning walks (reLWs). **a**, **b** Gaze directions of novices during iLW pirouettes (**a**, *n* = 14) and of foragers during reLW pirouettes (**b**, *n* = 10). Data is plotted in gray and the corresponding statistics in red. The bins include 10°. The red circle indicates the significance level of the Rayleigh uniformity test (*α* = 0.05). The red arrow is the r-vector pointing to the mean gaze direction. If the vector exceeds the red circle, data is directed significantly. In that case, the 95% confidence (95% CI) interval is shown as a red line outside the circle. If the expected direction (nest = 180°) lies within the 95% CI limits, data is directed towards the nest entrance. The outer circle indicates tic 3. **c** Proportion of novices (*n* = 15) and foragers (*n* = 10) that returned to the nest (black) or left the recording area (white). Fisher’s Exact Test: *p* < 0.001. For further details, see text

The ants underwent a characteristic ontogeny of learning-walk behavior and foraging trips (for one example of an individual ant: (Fig. 3). After several LWs (Fig. 3a, b), the ants moved further away, their paths straightened, and they became faster (Fig. 3c, d). Changes in the panorama, e.g. by an artificial landmark next to the nest entrance, induced reLWs (Fig. 3e, f). There were two main differences between iLWs by novices and reLWs by experienced foragers (Fig. 4). First, even though in both LW types pirouettes occurred, the ants’ gaze directions during the longest stopping phases were only directed towards the nest entrance (defined as 180°) during iLWs (Rayleigh Uniformity Test: *Z* = 3.242, *n* = 14, *p* < 0.05, *µ* = 171.7°, 95% CI (−/+): 127.7°/215.8°; Fig. 4a. Note that in that case the 95% CI limits may be unreliable, because of low concentration). In contrast, the gaze directions during the longest stopping phase of reLW pirouettes were randomly distributed (Rayleigh Uniformity Test: *Z* = 0.289, *n* = 10, *p* = 0.758, Fig. 4b). There was also no tendency that the ants gazed at the landmark, but gaze directions during the longest stopping phase of reLW pirouettes relative to the position of the landmark were randomly distributed (Rayleigh Uniformity Test: *Z* = 0.131, *n* = 10, *p* = 0.882). The gaze directions relative to the nest entrance during iLWs and reLWs differed significantly (Mardia–Watson–Wheeler Test: *n*_novices_ = 14, *n*_foragers_ = 10, *W* = 6.653, *p* < 0.05).

The second clear difference between iLWs and reLWs was that novices performing iLWs returned usually directly to the nest (13 of 15 novices), whereas foragers always ran off on a foraging trip after performing a reLW (*n* = 10) (Fisher’s Exact Test: *p* < 0.001, Fig. 4c).

### Magnetosensation in re-learning walks of foragers

**Fig. 5:**
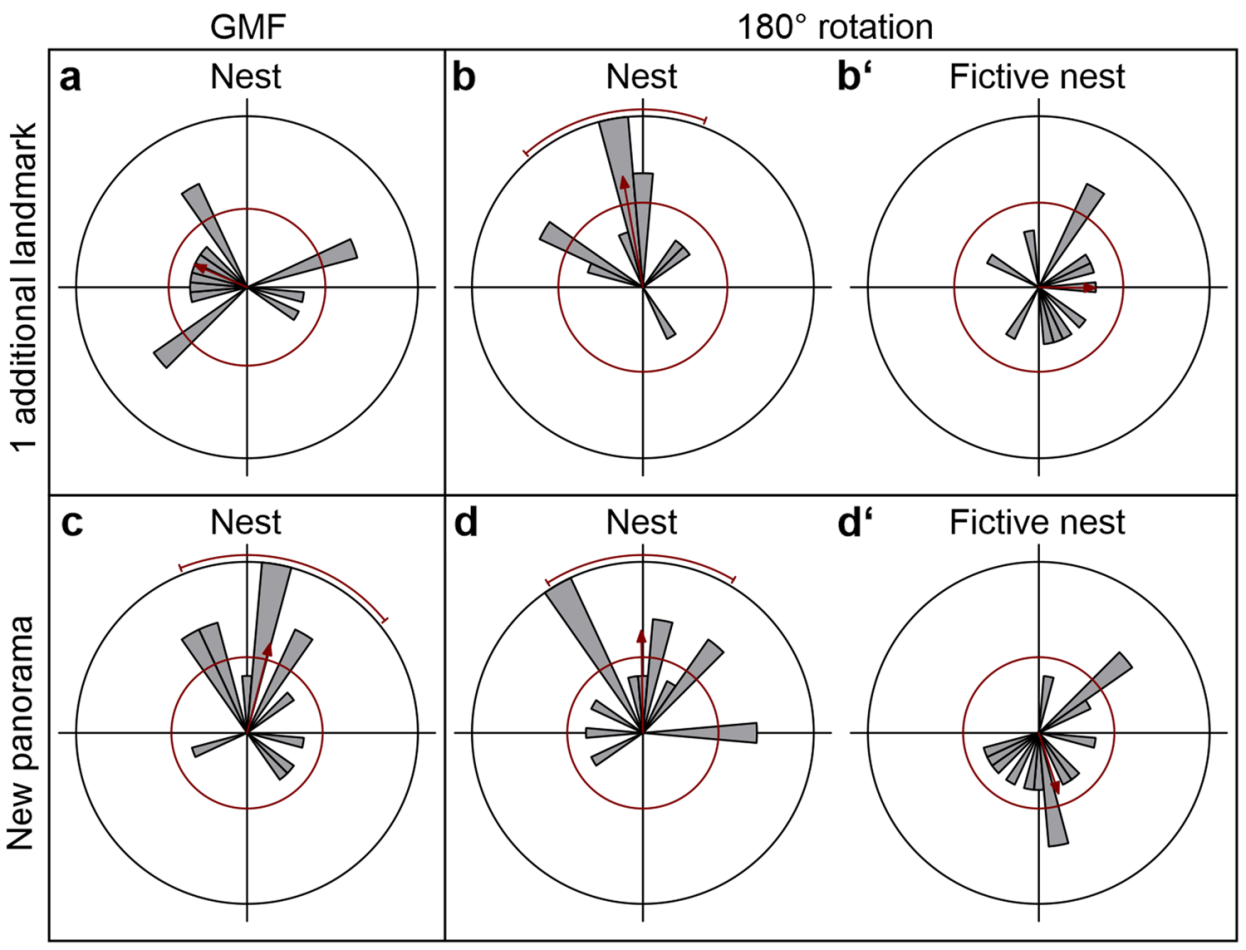
Gaze directions of foragers before and after 180° rotation of the horizontal component of the magnetic field. For figure conventions, see Fig. 4. **a–b′** Gaze directions of foragers during reLW pirouettes when one additional landmark had been installed **a** *n* = 14, **b**, **b′** *n* = 12. **c–d′** Gaze directions of foragers during reLW pirouettes when they were confronted with a new panorama (*n* = 15. **a**, **c** Gaze directions relative to the nest entrance before magnetic alteration (under natural geomagnetic field, GMF). **b**, **d** Gaze directions relative to the nest entrance after magnetic alteration. **b′**, **d′** Same data as in b, d) plotted relative to the fictive nest entrance

Since experienced foragers had performed pirouettes during reLWs, we conducted the second experiment to investigate the role of the GMF during reLWs. Experienced foragers only gazed back to the nest entrance when the panorama had been changed drastically (Fig. 5a, c). When foragers were used to the setup and a single landmark had been placed on the experimental table (Fig. 1b), they did not gaze back to the nest entrance during reLW pirouettes (Rayleigh Uniformity Test: *Z* = 2.091, *n* = 15, *p* = 0.123, Fig. 5a). In contrast, when foragers were confronted with a new panorama (Fig. 1c), they gazed back to the nest entrance during reLW pirouettes (Rayleigh Uniformity Test: *Z* = 4.352, *n* = 15, *p* < 0.05, *µ* = 194.4°, 95% CI (−/+): 158.0°/230.8°, Fig. 5c). When the Helmholtz coil was switched on to turn the horizontal component of the magnetic field by 180°, foragers always gazed back to the real nest entrance (Fig. 5b, d), but not back to the fictive nest entrance position predicted by the 180° turn of the horizontal component of the magnetic field (Fig. 5b′, d′). Under both experimental conditions A (one additional landmark) and B (new panorama), foragers gazed back to the real nest entrance (Rayleigh Uniformity Test: one additional landmark: *Z* = 5.197, *n* = 12, *p* < 0.01, *µ* = 169.6°, 95% CI (−/+): 138.8°/200.5°, Fig. 5b; new panorama: *Z* = 5.371, *n* = 15, *p* < 0.01, *µ* = 179.1°, 95% CI (−/+): 147.6°/210.6°, Fig. 5d). They did not gaze back to the fictive nest entrance position indicated by the experimental magnetic field (Rayleigh Uniformity Test: one additional landmark: *Z* = 1.318, *n* = 12, *p* = 0.273, Fig. 5b′; new panorama: *Z* = 2.091, *n* = 15, *p* = 0.123, Fig. 5d′). The gaze directions of the foragers confronted with one additional landmark before and after the magnetic alteration were different (Mardia–Watson–Wheeler Test: one additional landmark: *n*_before_ = 14, *n*_after_ = 12, *W* = 6.562, *p* < 0.05). The gaze directions of the foragers with the new panorama did not change significantly after alteration of the magnetic field (Mardia–Watson–Wheeler Test: new panorama: *n*_before_ = 15, *n*_after_ = 15, *W* = 0.106, *p* = 0.948).

## Discussion

Experienced ant foragers perform a conspicuous behavior—so-called re-learning walks (reLWs)—when the panorama around the nest entrance has been changed (Fleischmann et al. 2016; Jayatilaka et al. 2018; Müller and Wehner 2010; Narendra and Ramirez-Esquivel 2017; Vega Vermehren et al. 2020; Zeil and Fleischmann 2019). Our results show that reLWs of experienced foragers can be clearly distinguished from initial learning walks (iLWs) of novices due to several characteristics.

Most obviously, novices return to the nest at the end of an iLW, whereas foragers run off to pursue a foraging trip after the reLW. Similar results have been obtained with honeybees showing that re-orienting foragers cover wider areas than inexperienced honey bees during their initial exploratory flights (Degen et al. 2018). Remarkably, *C. nodus* ants usually circle around the nest entrance covering almost every sector before pursuing a foraging trip, as do other ant species. For example, Namibian desert ants (Müller and Wehner 2010) and Australian jack jumper ants (Jayatilaka et al. 2018) show spiral-shaped reLWs, too. Another interspecific similarity is that both *C. nodus* and *M. croslandi* (Jayatilaka et al. 2018) meander during reLWs.

iLWs are necessary to acquire information for becoming successfully navigating foragers (Fleischmann et al. 2016, 2018b; Wehner et al. 2004). The ant brain, particularly synaptic relay stations in high-order integration centers along two visual pathways, undergoes plastic changes during the iLW phase (Grob et al. 2017, 2019; Rössler 2019; Stieb et al. 2012). Furthermore, the transition from interior to exterior worker is accompanied by changes in the expression levels of neuropeptides playing an important role in behavior control (Habenstein et al. 2021). This neuroplasticity supports the fact that novices perform visual learning and calibrate their visual guidance systems to acquire all information for orientation during foraging, e.g. learning visual landmarks (panorama). This may also include olfactory and tactile cues. In contrast, reLWs most likely serve updating previously memorized information about the visual surroundings. Futures studies are necessary to understand which neuronal changes are associated with re-learning processes.

Furthermore, the structures of iLWs and reLWs differ. *C. nodus* novices perform two types of turns during their iLWs, i.e., voltes and pirouettes (Fleischmann et al. 2017). Voltes are small-radius walked circles without any stops directed toward the nest direction. We did not observe any voltes in reLWs of *C. nodus* foragers. We hypothesize that voltes are performed to calibrate the celestial compass at the beginning of the foraging career (Fleischmann et al. 2017), and therefore, foragers may not have to perform voltes during reLWs. Furthermore, both iLWs and reLWs contain pirouettes that can be full or partial turns about the ant’s body axis. *Cataglyphis* novices gaze back to the nest entrance during the longest stopping phase of a pirouette (Fleischmann et al. 2017; Wehner et al. 2004). Remarkably, *C. nodus* novices do not use celestial cues as directional information to align their gaze directions towards the nest entrance (Grob et al. 2017). Instead, they use the GMF as a directional reference system for this task (Fleischmann et al. 2018a). In contrast, as we could show here, *C. nodus* foragers do not use the GMF to align their gaze directions during reLW pirouettes.

Foragers only gaze back to their nest entrance, when the panorama has been changed drastically, or when the horizontal component of the magnetic field has been additionally turned by 180°. This suggests that foragers gaze to the nest entrance when different orientation cues are in conflict. In experiment 2 A (one additional landmark), foragers gazed back to the nest entrance during reLW pirouettes only when the magnetic field was altered, too (conflict between magnetic field and celestial cues + known panorama). In experiment 2B (new panorama), foragers gazed back to the nest entrance under unaltered magnetic conditions (conflict between unknown panorama and natural celestial cues + GMF). They also gazed back to the nest entrance when the magnetic field was additionally rotated by 180° (conflict between natural celestial cues and unknown panorama + experimentally altered magnetic field). We, therefore, conclude that foragers are magnetosensitive, but do not use the magnetic field as a reference system for aligning their nest-directed gazes. As the ants have already learned the celestial compass cues during iLWs, experienced foragers most likely keep relying on celestial compass information, to gaze back to the nest entrance at this stage of their ontogeny.

## Abbreviations

CI: Confidence interval
GMF: Geomagnetic field
iLW: Initial learning walk
LW: Learning walk
PI: Path integration
reLW: Re-learning walk

## References

[CR1] Boles LC, Lohmann KJ (2003). True navigation and magnetic map in spiny lobsters. Nature.

[CR2] Degen J, Hovestadt T, Storms M, Menzel R (2018). Exploratory behavior of re-orienting foragers differs from other flight patterns of honeybees. PLoS One.

[CR3] Dreyer D, Frost B, Mouritsen H, Günther A, Green K, Whitehouse M, Johnsen S, Heinze S, Warrant E (2018). The Earth’s magnetic field and visual landmarks steer migratory flight behavior in the Nocturnal Australian Bogong Moth. Curr Biol.

[CR4] Fleischmann PN, Christian M, Müller VL, Rössler W, WehnerR, (2016). Ontogeny of learning walks and the acquisition of landmark information in desert ants, *Cataglyphis fortis*. J Exp Biol.

[CR5] Fleischmann PN, Grob R, Wehner R, Rössler W (2017). Species-specific differences in the fine structure of learning walk elements in *Cataglyphis* ants. J Exp Biol.

[CR6] Fleischmann PN, Grob R, Müller VL, Wehner R, Rössler W (2018). The geomagnetic field is a compass cue in *Cataglyphis* ant navigation. Curr Biol.

[CR7] Fleischmann PN, Rössler W, Wehner R (2018). Early foraging life: spatial and temporal aspects of landmark learning in the ant *Cataglyphis noda*. J Comp Physiol A.

[CR8] Fleischmann PN, Grob R, Rössler W (2020). Magnetoreception in Hymenoptera: importance for navigation. Anim Cogn.

[CR9] Grob R, Fleischmann PN, Grübel K, Wehner R, Rössler W (2017). The role of celestial compass information in *Cataglyphis* ants during learning walks and for neuroplasticity in the central complex and mushroom bodies. Front Behav Neurosci.

[CR10] Grob R, Fleischmann PN, Rössler W (2019). Learning to navigate—how desert ants calibrate their compass systems. Neuroforum.

[CR11] Grob R, El-Jundi B, Fleischmann PN (2021). Towards a common terminology for arthropod spatial orientation. Ethol Ecol Evol.

[CR12] Guerra PA, Gegear RJ, Reppert SM (2014). A magnetic compass aids monarch butterfly migration. Nat Commun.

[CR13] Habenstein J, Thamm M, Rössler W (2021). Neuropeptides as potential modulators of behavioral transitions in the ant *Cataglyphis nodus*. J Comp Neurol.

[CR14] Jayatilaka P, Murray T, Narendra A, Zeil J (2018). The choreography of learning walks in the Australian jack jumper ant *Myrmecia croslandi*. J Exp Biol.

[CR15] Lohmann K, Lohmann CMF, Ehrhart LM, Bagley DA, Swing T (2004). Geomagnetic map used in sea-turtle navigation. Nature.

[CR16] Mouritsen H (2018). Long-distance navigation and magnetoreception in migratory animals. Nature.

[CR17] Müller M, Wehner R (1988). Path integration in desert ants, *Cataglyphis fortis*. Proc Natl Acad Sci.

[CR18] Müller M, Wehner R (2010). Path integration provides a scaffold for landmark learning in desert ants. Curr Biol.

[CR19] Narendra A, Ramirez-Esquivel F (2017). Subtle changes in the landmark panorama disrupt visual navigation in a nocturnal bull ant. Philos Trans R Soc B Biol Sci.

[CR20] Nicholson D, Judd SPD, Cartwright BA, Collett TS (1999). Learning walks and landmark guidance in wood ants (*Formica rufa*). J Exp Biol.

[CR21] Putman NF, Williams CR, Gallagher EP, Dittman AH (2020). A sense of place: Pink salmon use a magnetic map for orientation. J Exp Biol.

[CR22] Quinn TP (1980). Evidence for celestial and magnetic compass orientation in lake migrating sockeye salmon fry. J Comp Physiol A.

[CR23] Rössler W (2019). Neuroplasticity in desert ants (Hymenoptera: Formicidae)—importance for the ontogeny of navigation. Myrmecol News.

[CR24] Schmid-Hempel P, Schmid-Hempel R (1984). Life duration and turnover of foragers in the ant *Cataglyphis bicolor* (Hymenoptera, Formicidae). Insectes Soc.

[CR25] Schmitt F, Stieb SM, Wehner R, Rössler W (2016). Experience-related reorganization of giant synapses in the lateral complex: potential role in plasticity of the sky-compass pathway in the desert ant *Cataglyphis fortis*. Dev Neurobiol.

[CR26] Stieb SM, Hellwig A, Wehner R, Rössler W (2012). Visual experience affects both behavioral and neuronal aspects in the individual life history of the desert ant *Cataglyphis fortis*. Dev Neurobiol.

[CR27] Vega Vermehren JA, Buehlmann C, Fernandes ASD, Graham P (2020). Multimodal influences on learning walks in desert ants (*Cataglyphis fortis*). J Comp Physiol A.

[CR28] Wehner R (2020). Desert navigator: the journey of an ant.

[CR29] Wehner R, Meier C, Zollikhofer C (2004). The ontogeny of foraging behaviour in desert ants, *Cataglyphis bicolor*. Ecol Entomol.

[CR30] Wiltschko W, Wiltschko R (2005). Magnetic orientation and magnetoreception in birds and other animals. J Comp Physiol A.

[CR31] Wyeth RC (2010). Should animals navigating over short distances switch to a magnetic compass sense?. Front Behav Neurosci.

[CR32] Zeil J, Fleischmann PN (2019). The learning walks of ants (Hymenoptera: Formicidae). Myrmecol News.

